# Advanced Nanomaterials for Biological, Medical, and Environmental Applications: Current Progress and Future Perspectives

**DOI:** 10.3390/ma19183841

**Published:** 2026-09-09

**Authors:** Miruna S. Stan

**Affiliations:** Department of Biochemistry and Molecular Biology, Faculty of Biology, University of Bucharest, 91-95 Splaiul Independentei, 050095 Bucharest, Romania; miruna.stan@bio.unibuc.ro

## 1. Introduction and Special Issue Scope

Nanotechnology continues to be one of the most dynamic and transformative areas of modern science. Advances in nanomaterial synthesis, surface engineering, characterization techniques, and computational modeling have enabled the development of increasingly sophisticated nanosystems for applications ranging from medicine [1] and biotechnology to environmental monitoring [2] and remediation [3]. The ability to tailor physicochemical properties at the nanoscale has facilitated the creation of materials with enhanced functionality, selectivity, and responsiveness to external stimuli.

In the biomedical field, nanomaterials have demonstrated considerable potential [4] as drug delivery vehicles, imaging agents, biosensors, and therapeutic platforms. Simultaneously, environmental applications have expanded rapidly, with nanomaterials being employed for pollutant detection, water treatment, environmental monitoring, and sustainability-oriented technologies. More recently, the integration of artificial intelligence (AI), machine learning, and advanced analytical tools has opened new opportunities for accelerating nanomaterial discovery and improving the interpretation of complex experimental data [5].

Despite these advances, important challenges remain. The increasing complexity of multifunctional nanosystems necessitates interdisciplinary approaches that combine materials science, biology, environmental science, engineering, and data science.

The Special Issue “Advanced Nanomaterials for Biological, Medical and Environmental Applications (3rd Edition)” was launched to provide a platform for presenting recent developments addressing these challenges. The contributions collected in this issue illustrate the diversity of contemporary nanomaterial research while highlighting several emerging directions that are likely to shape the field in the coming years.

## 2. Emerging Themes from the Special Issue

One important area concerns the development of nanomaterials for therapeutic applications. Kim et al. [1] developed a near-infrared (NIR)-responsive microbubble platform that enables externally triggered drug release, highlighting the growing interest in stimuli-responsive nanocarriers for precision medicine. Such systems may improve therapeutic efficacy while minimizing adverse effects through localized and controlled drug delivery [6].

Other contributions included in this Special Issue further demonstrate the importance of magnetic nanomaterials for hyperthermia applications and multifunctional sustained-release nanosystems for antifungal delivery. These studies emphasize the increasing complexity of therapeutic nanoplatforms and their potential for targeted and personalized treatment strategies.

Environmental applications also represent a major research direction. Zhao et al. [2] developed a highly sensitive surface-enhanced Raman spectroscopy (SERS) substrate combined with deep-learning algorithms for microplastic detection, demonstrating the advantages of integrating nanotechnology with artificial intelligence. Such approaches offer promising opportunities for environmental monitoring, pollutant detection, and risk assessment.

Sustainability considerations are increasingly incorporated into nanomaterial development. Stoica et al. [3] combined material synthesis, ecotoxicological evaluation, and life-cycle assessment, emphasizing the importance of balancing material performance with environmental impact. This work reflects the growing interest in safe and sustainable-by-design materials and the implementation of circular economy principles.

## 3. Future Perspectives

Despite significant advances, several important challenges remain before advanced nanomaterials can achieve widespread clinical, industrial, and environmental implementation. Future research should therefore focus on improving reproducibility, scalability, manufacturing processes, and regulatory acceptance [7]. The development of standardized characterization protocols and harmonized testing methodologies will be essential for facilitating technology transfer and improving data comparability [8].

Artificial intelligence and machine learning are expected to play an increasingly important role in nanotechnology. AI-assisted approaches may accelerate nanomaterial design, predict biological responses, optimize synthesis conditions, and support toxicity assessment [9,10,11]. The successful integration of deep learning with nanostructured sensing systems reported in this Special Issue illustrates the considerable potential of these methodologies.

Comprehensive safety evaluation remains another major priority [12]. Recent advances in nanotoxicology emphasize the importance of physiologically relevant models, advanced in vitro systems, organ-on-chip technologies, and computational toxicology approaches [7,11]. Future studies should investigate long-term biological interactions, biodistribution, immunological responses, and environmental fate to ensure the safe implementation of nanotechnologies.

The development of environmentally friendly nanomaterials also represents a growing research direction. Green synthesis approaches, renewable resources, biodegradable materials, and life-cycle assessment methodologies should become integral components of nanomaterial development [13]. The implementation of safe and sustainable-by-design principles may contribute to reducing environmental impacts while maintaining material performance [14].

Finally, the complexity of contemporary challenges requires close collaboration among materials scientists, chemists, biologists, toxicologists, clinicians, environmental scientists, engineers, and data scientists. Interdisciplinary research will continue to be essential for translating fundamental discoveries into practical applications and for addressing global health and environmental challenges.

## 4. Conclusions

The papers collected in this Special Issue demonstrate the remarkable interdisciplinary nature of contemporary nanomaterials research. From controlled drug delivery and magnetic hyperthermia to environmental sensing, microplastic detection, and sustainable material design, these contributions illustrate how advanced nanomaterials continue to expand their impact across biological, medical, and environmental domains. At the same time, they highlight the importance of addressing critical challenges related to safety, sustainability, reproducibility, and translation into practical applications. I truly hope that this Special Issue will stimulate further interdisciplinary collaborations and inspire future research aimed at realizing the full potential of advanced nanomaterials in addressing global health and environmental challenges.
